# Emotional Attractors in Subject-Verb Number Agreement

**DOI:** 10.3389/fpsyg.2022.880755

**Published:** 2022-06-29

**Authors:** Anna Hatzidaki, Mikel Santesteban

**Affiliations:** ^1^Department of English Language and Literature, National and Kapodistrian University of Athens, Athens, Greece; ^2^Department of Linguistics and Basque Studies, University of the Basque Country (UPV/EHU), Vitoria-Gasteiz, Spain

**Keywords:** number agreement, comprehension, attraction effect, emotional word processing, ERPs

## Abstract

Considering the crosstalk between brain networks that contain linguistic and emotional information and that no studies have examined the impact of semantic information of affective nature on subject-verb number agreement, the present Event Related Potential (ERP) study investigated the extent to which emotional local nouns whose number mismatched that of subject head nouns might be considered by the parser during comprehension of grammatically correct sentences. To this end, twenty-eight Spanish native speakers were tested on a self-paced reading task while their brain activity was recorded. The experimental materials consisted of 120 sentences where the valence (negative vs. neutral) and number (singular vs. plural) of the local noun of the singular subject noun-phrase (NP) were manipulated; *El gorro de aquel/aquellos cazador(es)/mecánico(s) era*… [The hat of that/those hunter(s)/mechanic(s) was…]. ERP results measured in the local noun position showed that valence and number interacted in the 300–500 ms (negative component) and 780–880 ms (late positivity) time windows. In the (target) verb position, the two factors only interacted in the late 780–880 ms time window, revealing an “ungrammatical illusion” for plural marked neutral words. Our findings suggest that number agreement is sensitive to affective meaning but that the emotional information of an attractor is considered in different operations and at different stages during grammatical sentence processing; it can affect lexical and syntactic representation retrieval of a subject-NP and impact agreement encoding only at late stages of processing, during verb agreement and feature integration.

## Introduction

Subject-verb number agreement in Spanish, as in many other languages, conforms to the rule of having the number morphological features of the verb agreeing with those of the subject noun-phrase (NP) (see Acuña-Fariña, 2009 for a review). However, findings from the psycholinguistic literature have shown that agreement is not susceptible to influences coming neither only from the head noun nor only from syntax. For example, number agreement can be affected by factors, such as the difference between the morphological singular number of the head nouns and the morphological plural number of the closest-to-the-verb (local) noun (Bock and Miller, 1991; Haskell and MacDonald, 2003; Bock and Middleton, 2011) or the distance between a mismatching feature embedded in a prepositional phrase (PP) and the subject head noun (e.g., Franck et al., 2002). Evidence showing that agreement does not come entirely from a syntactic source and that it is sensitive to the role of semantics comes from studies showing that plural local nouns elicit more plural agreement attraction errors when the noun phrases have a notional distributive reading (e.g., *The label on the bottles*) than when they do not (e.g., The house of my cousins; Vigliocco et al., 1995, 1996). These findings suggest that grammatical information of a subject can be overridden by the number specification of the conceptual representation of the subject phrase and that conceptual factors may impact grammatical encoding. In all the above cases, the observed result is that the verb seems to be “attracted” to the plural number of the preceding noun when it mismatches the number of the head noun. This may happen mainly for three reasons: (i) the plural feature of the local noun may override the default assignment by being mistakenly detected by the verb-agreement mechanism (*feature percolation account*; Bock and Eberhard, 1993); (ii) singular number (being morphologically unmarked) is more vulnerable to the influence of plural number (Eberhard, 1997); or (iii) due to unsuccessful number reconciliation of the number features of the subject noun when agreement features are transmitted to the target verb (*marking and morphing account*; Bock et al., 2001).

In comprehension studies, attraction effects have been demonstrated mainly in the form of “grammatical illusions.” Thus, sentences, such as “The key to the cabinets *are …,” seem to be processed faster, as shown in reaction time (e.g., Wagers et al., 2009) and as if they are grammatically correct, as demonstrated in the reduced negative left anterior negativity (LAN/N400) or positive (P600) components of agreement violation detection as compared to other ungrammatical sentences, such as “The key to the cabinet *are…” (e.g., Tanner and Van Hell, 2014). Such illusions have also been explained by a feature percolation account that assumes erroneous percolation into a subject noun position when syntactic constituents are hierarchically integrated into a processing structure (e.g., Bock and Cutting, 1992). In addition, they have been accounted for by a cue-based retrieval failure during syntactic integration caused by feature slippage or misidentification of the correct controller of agreement (e.g., Wagers et al., 2009).

However, there are findings to suggest that even in grammatical sentences, effects of attraction can be shown in the form of “ungrammatical illusions.” Nicol et al. (1997) found slower reading times and less accurate responses when a singular head noun was followed by a plural than a singular attractor, as in “The author of the speeches is…” vs. “The author of the speech is…” in a Maze task where participants had to decide which of the two words was a better continuation for a sentence. Laurinavichyute and von-der-Malsburg (2019) found that a plural noun that mismatched the number of a subject noun in grammatical sentences, such as “The admirer of the singers supposedly thinks that…” slowed down processing on the verb. Processing difficulty in correct sentences of NP-mismatch conditions with a singular head (Experiment 3: *The key to the cabinets was*…), as displayed in slower reading times, was also reported by Pearlmutter et al. (1999). Similarly, Franck et al. (2015) showed that plural object interveners slowed down the grammaticality judgment of subject-verb dependencies. In an Event Related Potential (ERP) study, Kaan (2002) found that the grammatical condition of a singular subject and a plural object in German yielded an enhanced early positivity at the critical verb. In another ERP study, Martin et al. (2012) found evidence of processing costs during comprehension of elliptical sentences, such as “*Marta se compró la camiseta que estaba al lado del vestido y Miren cogió otra*…” (Marta bought the t-shirt_[*FEM*]_ that was next to the dress_[*MASC*]_ and Miren took another_[*FEM*]_…): the gender of a mismatching attractor emitted larger negativity and larger late positivity in the condition where the attractor had a different gender from the antecedent. In other words, when the attractor did not match the retrieval cue, this had an impact on the processing of grammatical sentences as well. Finally, in a sentence completion study in Basque, the only study where the number of attraction effects have been examined in grammatical sentences at an electrophysiological level, Santesteban et al. (2020) found slower subject-verb production when the subject and object mismatched in number than when they matched. Mismatching objects elicited an early production P2 followed by a negative component, showing the difficulty of number feature retrieval and monitoring during correct subject-verb agreement production.

Recent studies that have looked at the impact of semantic factors on agreement processing have considered the case of emotional meaning. This is not surprising as emotional content due to its salience, with affective information being prioritized over non-affective information and capturing attention resources (e.g., Zajonc, 1980, 2001; Delaney-Busch and Kuperberg, 2013), has been found to affect lexical processing and shows its signature in neural implementation as well. More enhanced effects are reported for emotional than non-emotional words (e.g., knife vs. sink) whether they are processed in isolation or embedded in sentences and interact with other semantic or morphosyntactic information (see Kissler et al., 2006; Citron, 2012 and Hinojosa et al., 2020 for reviews). The majority of ERP studies at the sentential level that have examined the influence of emotional valence on the agreement have considered the case of gender agreement in Spanish (e.g., Hinojosa et al., 2014; Díaz-Lago et al., 2015; Fraga et al., 2017; Jiménez-Ortega et al., 2017). Some have found that the detection of gender agreement violations between adjectives and nouns can be affected at the early stages of morphosyntactic processing by whether the content of the agreeing element is emotional, as reflected by the interaction between grammaticality and valence in the LAN/N400 time window (Hinojosa et al., 2014; Jiménez-Ortega et al., 2017; Fraga et al., 2021). With regard to number agreement and sentence comprehension with ERPs, which is the focus of the present study, to our knowledge, there are only two studies that have investigated the role of emotional words (Martín-Loeches et al., 2012; Jiménez-Ortega et al., 2017 where the manipulation of emotional information involved a subliminal presentation of adjectives as the question of interest was centered on the automaticity of syntactic processing). Here, we focus on the study by Martín-Loeches et al. (2012) because in their experimental procedure the variables of valence and number were manipulated supraliminally, as in our study. The researchers used Spanish sentences of determiner-noun-adjective-verb structure for syntactic processing (Experiment 1) and manipulated emotional valence (positive, negative, neutral adjectives) and grammaticality (syntactically correct vs. incorrect). Thus, the adjective, which was the critical word, either matched or mismatched the number of the noun it modified and in the latter case resulted in the creation of syntactic violations: e.g., *La hermana querida acude* (The loved_[SG]_ sister arrives) vs. *La hermana queridas acude (The loved_[PL]_ sister arrives); *La chica fea baila* (The ugly_[SG]_ girl dances) vs. *La chica feas baila (The ugly_[PL]_ girl dances); and *El espejo ovalado refleja* (The oval_[SG]_ mirror reflects) vs. *El espejo ovalados_[PL]_ *refleja*.

Event-Related Potentials elicited during the performance of a grammaticality judgment task revealed a larger LAN (350–450 ms) component for incorrect than correct sentences only when the adjective was negative rather than neutral. Thus, morphosyntactic processes were found to be sensitive to the emotional information carried by the syntactically anomalous emotional words and affect the detection of number agreement of violations between the adjective and its noun. A late positive component (P600; 600–700 ms) was elicited by ungrammatical sentences and was not modulated by valence, suggesting that emotional information modulated grammatical processing only at the early stages of agreement computation (reflected in the LAN component).

Despite the merits of the study of Martín-Loeches et al. (2012), some aspects of the materials and the design may not have offered the best condition for clear evidence of valence and morphosyntactic effects on number agreement (for a review of methodological and procedural issues that may have contributed to discrepancies in studies of emotional impact on gender and number agreement see Fraga, 2020). Apart from emotionality, animacy was different between the conditions of interest, as the subject noun in the emotional conditions (positive/negative) was animate, whereas in the neutral condition it was inanimate. Importantly, the critical word (the adjective) was the element that bore both the valence and agreement manipulations making rather difficult the attribution of effects. Finally, language use is predominantly based on computation and processing of well-formed utterances both in comprehension and in production, and given that the existing relevant literature has mainly considered syntactic violations, more research is needed to address number agreement processing in grammatically correct contexts.

Thus, in the present study, we sought to investigate the interplay between morphosyntactic and semantic information in number agreement not examined so far. Specifically, we investigated the extent to which the emotional content of a local noun, i.e., of an element not directly relevant to the computation of subject-verb number agreement but of reported salience, might be considered by the parser along with its morphosyntactic features and affect the application of syntactic rules for the comprehension of grammatical sentences. That is, we tested the effect of emotion under the most stringent agreement conditions. Our focus was on two ERP components because they are the most relevant ones for current purposes: a negative component/N400 between 300 and 500 ms that when yielded is associated with initial emotional analysis (e.g., Delaney-Busch and Kuperberg, 2013) and a late posterior positive component/late posterior positivity (LPP)/late positive component (LPC)/P600 (after 500 ms) that is typically associated with sustained attention to emotional input and elaborate processing (e.g., Bayer et al., 2010; see Hinojosa et al., 2020 for a review). Regarding syntactic computations of ERP components in the same time windows, a (left anterior) negativity has been suggested to index the processing of dependency relations and is emitted when morphosyntactic violations or mismatches are detected. A late (centroparietal) positivity is associated with effects of reanalysis of syntactic violations or of expectations (present study) of agreement relations, which are not consistent with the syntactic analysis taking place (e.g., Osterhout and Mobley, 1995; Kutas and Federmeier, 2011; see Kuperberg, 2007 and Molinaro et al., 2011 for reviews.) Importantly, to obtain a clear picture of the effect of emotional attractors as emotionally loaded words (on a purely semantic level) and as syntactic interfering elements (on a syntactic level), we performed separate analyses in the position of the attractor (local noun) and of the target (verb), respectively.

## Method

### Participants

In total, 28 Spanish native speakers (5 men, age *M* = 20.6; SD = 1.8), undergraduate students at the University of the Basque Country (UPV/EHU) received monetary compensation for their participation. The experiment was approved by the University ethics committee and all participants provided a signed consent prior to the experiment.

### Materials and Procedure

Experimental materials consisted of 120 grammatical sentences involving singular subjects with a PP modifier. Each sentence was presented in four experimental conditions (30 sentences per condition), as a result of crossing the manipulation of Valence (negative vs. neutral) and Number (singular vs. plural) of the attractor nouns inside the PP modifying the singular subject: *El gorro de aquel/aquellos cazador(es)/mecánico(s) era de gran colorido por seguridad* [The hat of that/those hunter(s)/mechanic(s) was very colorful for safety]. Half of the sentences had negative attractors and half neutral ones (taken from Davis and Perea, 2005; Redondo et al., 2007). All sentences were 11 words long and had an inanimate neutral subject noun and an animate attractor (in position 5). The verb (in position 6) was singular, as was the subject noun, since all sentences were grammatical. Negative and neutral attractors were controlled for frequency (*M* = 9.3 vs. *M* = 9.4 per million); length (*M* = 7.3 vs. *M* = 7.4); number of syllables (*M* = 3.2 vs. *M* = 3.2); and concreteness (*M* = 6.8 vs. *M* = 7.1; 1–7 scale); [all ts(118) < 1.98]. They only differed between them with regard to valence [*M* = 2.6 vs. *M* = 5.3; *t*(118) = 23.05, *p* < 0.001] and arousal [*M* = 5.8 vs. *M* = 4.5; *t*(118) = 8.34, *p* < 0.001; 1–9 scale]. An additional set of 120 filler sentences of subject and relative clauses were also included in the stimuli.

The experiment was performed using Presentation software (version 16.0^1^). Prior to the experiment, participants were instructed about the electroencephalogram (EEG) procedure and were seated comfortably in a quiet room in front of a 17-inch monitor. Sentences were displayed in the middle of the screen word-by-word for 350 ms interstimulus interval (ISI = 250 ms) in a serial visual presentation paradigm. Participants were asked to read the sentences silently for comprehension and answer YES/NO questions in 33% of the trials by pressing one of two keys on a keyboard placed on their lap [e.g., sentence: *La cita de la camarera era a las ocho y media* (The appointment of the waitress was at half past 8). Question: *>La cita era a las doce y media?* (Was the appointment at half past 12?)]. A fixation cross (+) was presented for 1,000 ms prior to each trial. Materials were pseudo-randomized so that no two sentences of the same condition were displayed one after the other and each experimental sentence was followed by a filler sentence. All 240 sentences were distributed over four blocks, allowing breaks in between. A short practice session of six trials preceded the experiment. Each session that included the easy-cap application and removal lasted about 1 h.

### Electroencephalogram Recording

The ERPs were recorded from 32 scalp electrodes mounted in an ActiCAP International (Inc.; 10–20 system). The electrodes were placed as follows: Fp1, Fp2, F7, F3, Fz, F4, F8, FC5, FC1, FC2, FC6, T7, C3, Cz, C4, T8, CP5, CP1, CP2, CP6, P7, P3, Pz, P4, P8, O1, Oz, and O2. All electrodes were referenced to the right mastoid and re-referenced offline to the left mastoid electrode. The vertical and horizontal electro-oculograms (VEOG and HEOG) were recorded from electrodes located below (VEOG) and at the outer canthus (HEOG) of the right eye. Electrode impedance was kept below 10 kΩ at all scalp and mastoid sites and at the eye electrodes. Gratton and Coles’ ocular correction was applied and the electrical signals were digitalized online at a rate of 250 Hz and filtered offline with a bandpass of 0.1–35 Hz (half-amplitude cutoffs). Head movements and other artifacts were manually removed.

## Results

### Scoring and Data Analysis

Average ERPs were computed for the emotional attractor (local noun), the verb (target) position, and each electrode. Segments were constructed from 200 ms before and 1,000 ms after the onset of the word that was the focus of analysis.^2^ The trials associated with each sentence were averaged for each participant. The 200 ms prior to the onset was also used as a baseline for all sentence-type comparisons. After the baseline correction, epochs with artifacts were rejected. Based on the literature and visual inspection of the data, 300–500 and 780–880 ms time windows were considered during the statistical analysis. After the stimuli were recorded and averaged, repeated-measures ANOVAs were carried out in three regions of interest (ROI) that were computed out of the 28 electrodes: frontocentral (Fp1, Fp2, F7, F3, Fz, F4, F8, FC5, FC1, FC2, and FC6), centroparietal (T7, C3, Cz, C4, T8, CP5, CP1, CP2, and CP6), and parieto-occipital (P7, P3, Pz, P4, P8, O1, Oz, and O2). Initial analyses that also included hemisphere (left vs. right) did not yield significant interactions with the manipulated variables of valence and number either in the attractor or in the verb position. Thus, repeated-measures ANOVAs were performed over the experimental manipulations, using three within-participant factors: valence (negative vs. neutral), number (singular vs. plural), and region (frontocentral vs. centroparietal vs. parieto-occipital). Effects of the Region factor were reported only when they interact with the experimental manipulations.

### Attractor (Local Noun) Position

#### 300–500 ms Time Window (Negativity/N400)

The analysis within 300 and 500 ms after participants had read the attractor yielded a significant main effect of valence, *F*(1, 27) = 7.21, *p* = 0.012, η^2^p = 0.211, with an increased amplitude for negative than for neutral attractors (*M* = − 0.49 vs. *M* = 0.28); a significant main effect of number *F*(1, 27) = 7.01, *p* = 0.013, η^2^p = 0.206, with an increased amplitude for singular than for plural attractors (*M* = − 0.40 vs. *M* = 0.19); and a significant region by valence interaction *F*(2, 54) = 4.12, *p* = 0.022, η^2^p = 0.132. Because the three-way interaction approached significance, *F*(2, 54) = 2.96, *p* = 0.060, η^2^p = 0.099, we followed up with analyses of the interaction between valence and number in each region and found that it was significant in the frontocentral region, with an increased amplitude for negative than for neutral attractors when they were singular, *t*(27) = 2.70, *p* = 0.012 and for singular versus plural when they were negative, *t*(27) = 2.81, *p* = 0.009.

#### 780–880 ms Time Window [Late Positivity/Late Posterior Positivity (Late Positive Component)/P600]

**FIGURE 1:**
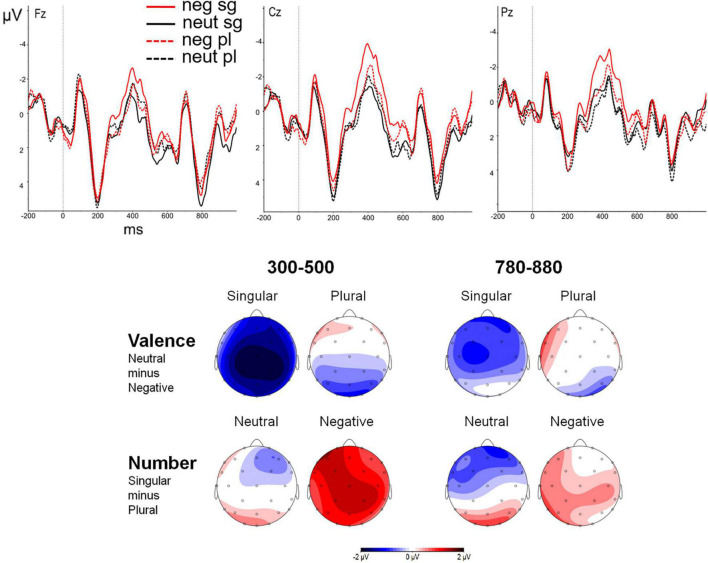
Event-Related Potentials (ERPs) to valence and number manipulations at the three midline electrodes (Fz, Cz, and Pz) and topographic maps in the attractor (local noun) position in 300–500 and 780–880 ms windows.

Analyses for the late positive component neither yielded a significant main effect of valence, *F*(1, 27) = 0.79, *p* = 0.380, η^2^p = 0.029, nor of number, *F*(1, 27) = 0.26, *p* = 0.613, η^2^p = 0.010. The interaction between region and number was significant, *F*(2, 54) = 5.51, *p* = 0.007, η^2^p = 0.169, as was the three way interaction between region, valence, and number, *F*(2, 54) = 3.56, *p* = 0.035, η^2^p = 0.117. Followed up analyses showed that the interaction between valence and number was significant in the frontal region, where there was a marginally significant attractor number effect for neutral attractors, *t*(27) = 1.84, *p* = 0.076, with an increased amplitude for singular than for plural attractors and a marginally significant valence effect in singular attractors, *t*(27) = 1.93, *p* = 0.064, with an increased amplitude for neutral than for negative attractors. [Figure 1 shows effects of valence and number of the attractor in the attractor (local noun) position in 300–500 and 780–880 ms windows, respectively.]

### Verb (Target) Position

#### 300–500 ms Time Window (Negativity/N400)

The analysis in the agreement position (of the verb) showed that the main effect of valence was not significant, *F*(1, 27) = 2.10, *p* = 0.159, η^2^p = 0.072, nor was the effect of number, *F*(1, 27) = 0.16, *p* = 0.691, η^2^p = 0.006. The interaction between region and valence was significant, *F*(2, 54) = 6.26, *p* = 0.004, η^2^p = 0.188, showing an increased amplitude for neutral than for negative attractors in the parieto-occipital region; *t*(27) = 2.09, *p* = 0.047.

#### 780–880 ms Time Window [Late Positivity/Late Posterior Positivity (Late Positive Component)/P600]

**FIGURE 2:**
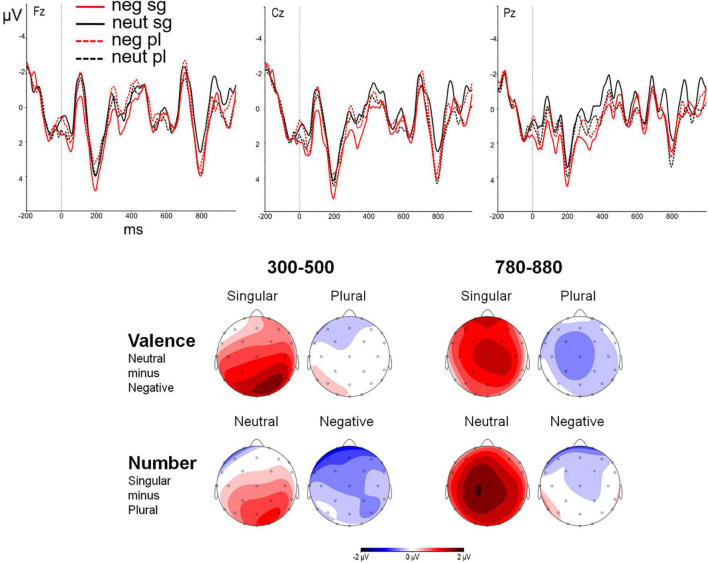
Event-Related Potential (ERPs) to valence and number manipulations at the three midline electrodes (Fz, Cz, and Pz) and topographic maps in the verb (target) position in 300–500 and 780–880 ms windows.

In the late time window, the analysis in the verb position did not show a significant main effect of valence, *F*(1, 27) = 0.72, *p* = 0.403, η^2^p = 0.026, nor of number, *F*(1, 27) = 2.49, *p* = 0.126, η^2^p = 0.084. The interaction between region and number approached significance, *F*(1, 27) = 2.62, *p* = 0.082, η^2^p = 0.089 and further analyses yielded a marginally significant difference between plural and singular attractors in the centroparietal region, *t*(27) = 1.94, *p* = 0.063, and a significant difference in the parieto-occipital region, *t*(27) = 2.32, *p* = 0.028, with plural attractors showing an increased amplitude when compared to singular attractors. The interaction between valence and number was marginally significant, *F*(1, 27) = 3.77, *p* = 0.063, η^2^p = 0.123, showing that valence effects were only present for singular attractors, with larger positivity for negative than for neutral attractors, *t*(27) = 2.08, *p* = 0.047. In addition, attractor number effects were only significant for neutral attractors, with larger positivity for plural than singular attractors, *t*(27) = 2.94, *p* = 0.007. (Figure 2 shows effects of valence and number of the attractor in the verb position in 300–500 and 780–880 ms windows, respectively.)

## Discussion

Considering the crosstalk between brain networks that contain linguistic and emotional information (Pulvermüller, 1999; Palazova, 2014; see Hinojosa et al., 2020 for a recent review) and the attention-grabbing effects that affective meaning appears to have on lexical processing (e.g., Delaney-Busch and Kuperberg, 2013), in the present ERP study, we explored the extent to which semantic information of affective nature may impact access to syntactic representations during subject-verb number agreement. Thus, we investigated the attraction effect of emotional local nouns, i.e., interference of structurally irrelevant items, on grammatical sentences during sentence comprehension.

Unlike previous studies on agreement dependencies, where the influence of emotional content was considered in tandem with morphosyntactic violations (Martín-Loeches et al., 2012; Hinojosa et al., 2014; Fraga et al., 2017), the design of the present study allowed us to tease apart semantic from syntactic effects and provide a clearer picture of the impact valence that may have on number agreement processing. The manipulation of valence (negative vs. neutral) and number (singular vs. plural) of the attractor showed different effects on the processing of the attractor as a lexical item (local noun) and on the way, the retrieval of the features of the attractor affected subject-verb agreement computation.

With regard to the former, as reflected in the negative ERP component that was yielded (300–500 ms), we found a strong valence effect only for singular attractors. The fact that the valence effect was absent for plural attractors may be due to the costlier processing of morphologically and semantically richer plural nouns, which might have canceled out the larger saliency of nouns with negative vs. neutral emotional valence. Thus, if plurality and valence effects were of similar size, they might have canceled each other out. Additionally, the fact that attractor nouns that matched in both number and valence with the (singular and neutral) subject noun tended to elicit a larger late positive ERP component (780–880 ms) suggests that the parser had more difficulty identifying the subject and discarding the attractor noun as a possible candidate to be assigned the subject role.

The effects shown on the agreeing verb are particularly interesting, as in verb position, one would expect to find the attraction effect in the form of an ungrammatical illusion and the impact of valence (if there was). The negative ERP component showed that subjecthood feature checking was costlier when the attractor had the same valence as the subject noun that was neutral. This suggests that head and attractor noun features were retrieved for agreement computation. Importantly, the late ERP positive component showed sensitivity to the emotional content of the local noun during feature integration and reanalysis. The fact that “ungrammatical illusions” caused by attraction on grammatical sentences (e.g., Franck et al., 2015) only showed with neutral attractors suggests that the saliency of emotionally negative attractors facilitated discarding the features of the attractor as the agreement source.

Despite being an underinvestigated topic, studying emotional effects in agreement, especially attraction effects in grammatical sentences, can have important implications. It can provide information about what is considered by the parser during online the processing of agreement relations and offer explanations about whether “ungrammatical illusions” can be attributed to the faulty mental encoding of linguistic representations or difficulty in accessing the right morphosyntactic information due to factors, such as the emotional value of an attractor. It can also shed light on the debate between strongly modular models that assume distinct sequential processes between syntactic and semantic representations (e.g., Ferreira and Clifton, 1986; Friederici and Weissenborn, 2007) and fully interactive models that assume that syntactic and semantic constraints interact simultaneously at the message-level representation (e.g., Hagoort, 2003) or intermediate accounts (e.g., Kim and Osterhout, 2005). Our findings suggest that number agreement is not insensitive to affective meaning but that emotional information of an attractor is considered in different operations and at different stages during grammatical sentence processing: for the retrieval of lexical and syntactic representations of the subject-NP and during subject-verb number agreement. Regarding number agreement processing, valence seems to be considered at an early stage of feature checking, where it acts as a cue for the selection of agreeing elements (a local noun with the same valence as the subject noun triggered similarity-based interference). At a late stage of reanalysis, both valence and number features of the attractor are checked to confirm grammaticality.

## Data Availability Statement

The raw data supporting the conclusions of this article will be made available by the authors, without undue reservation.

## Ethics Statement

The study was reviewed and approved by the Ethics Committee of the UPV/EHU (Comité de Ética para las Investigaciones relacionadas con Seres Humanos, CEISH; Project code: M10_2019_167). Participants provided their written informed consent to participate in this study.

## Author Contributions

AH and MS conceived and designed the study, collected the data, performed the statistical analyses, and drafted the manuscript. Both authors agreed on the final content prior to submission.

## Conflict of Interest

The authors declare that the research was conducted in the absence of any commercial or financial relationships that could be construed as a potential conflict of interest.

## Publisher’s Note

All claims expressed in this article are solely those of the authors and do not necessarily represent those of their affiliated organizations, or those of the publisher, the editors and the reviewers. Any product that may be evaluated in this article, or claim that may be made by its manufacturer, is not guaranteed or endorsed by the publisher.

## Footnotes

1
www.neurobs.com

2 As the purpose of the comprehension task that was used in our study was simply to ensure that participants were actually processing the displayed sentences, in the main text we focus on the analyses of the electrophysiological data. The accuracy (in%) of the participants was overall very high in all conditions: Negative-singular = 92.5 (8.4); Negative-plural = 93.2 (7.7); Neutral-singular = 91.4 (SD = 9.7); Neutral-plural = 90.7 (10.1). Repeated-measures ANOVA analyses did not show any significant effect (all Fs < 1). The response times of correctly answered questions were the following per condition: Negative-singular = 2,472 (441); Negative-plural = 2,575 (602); Neutral-singular = 2,477 (549); and Neutral-plural = 2,396 (452). The analyses only showed a marginally significant valence effect [*F*(1, 27) = 4.121, *p* = 0.052], η^2^p = 0.132, with a tendency for slower responses to questions about sentences containing negative (2,544 ms) than neutral (2,437 ms) attractor nouns.
