# Supplementary material for: Emotional Attractors in Subject-Verb Number Agreement
Source: Front Psychol. 2022 Jun 29;13:880755. doi: 10.3389/fpsyg.2022.880755 (PMC9330507; doi:10.3389/fpsyg.2022.880755)
Supplement: Supplementary file 1 [file Data_Sheet_1.docx]

**APPENDIX**

(Experimental materials used in the present study and their English translation in brackets.)

1. La leyenda de la(s) cigüeña(s)/tarántula(s) era muy común en esta comunidad.

(The legend of the stork(s)/tarantula(s) was very common in this community.)

2. El cuadro con el/los profesor(es)/dictador(es) era propiedad de aquel pequeño museo.

(The picture of the teacher(s)/dictator(s) was owned by that small museum.)

3. El proyecto para el/los peón(es)/preso(s) tenía aspectos técnicos de mucha dificultad.

(The project for the pawn(s)/prisoner(s) had very difficult technical aspects.)

4. La cita de la(s) camarera(s)/prostituta(s) era a las ocho y media.

(The appointment of the waitress(s)/prostitute(s) was at half past eight.)

5. El traje para el/los sastre(s)/viudo(s) era de los primeros años sesenta.

(The suit for the tailor(s)/widower(s) was from the early sixties.)

6. La plataforma para la(s) lechuza(s)/mofeta(s) podía sostener varios kilos de peso.

(The platform for the owl(s)/skunk(s) could support several kilos of weight.)

7. La charla sobre la(s) foca(s)/ostra(s) era generalmente muy interesante e informal.

(The talk about the seal(s)/oyster(s) was generally very interesting and informal.)

8. La aventura de la(s) cebra(s)/hiena(s) salió en el cine el viernes.

(The adventure of the zebra(s)/hyena(s) came out in theaters on Friday.)

9. El espejo con el/los grillo(s)/tábano(s) parecía arte moderno de nuestro siglo.

(The mirror with the cricket(s)/gadfly(s) looked like modern art from our century.)

10. La libreta de la(s) jardinera(s)/madrastra(s) tenía información importante y muy útil.

(The notebook of the gardener(s)/stepmother(s) had important and very useful information.)

11. La sombra de la(s) jirafa(s)/víbora(s) ocupaba más espacio de lo esperado.

(The shadow of the giraffe(s)/viper(s) took up more space than expected.)

12. La llamada para la(s) arquitecta(s)/drogadicta(s) llegó muy tarde para cambiar algo.

(The call for the architect(s)/drug addict(s) came too late to change anything.)

13. El archivo sobre el/los luchador(es)/violador(es) reveló muchos secretos al mundo entero.

(The file on the wrestler(s)/rapist(s) revealed many secrets to the whole world.)

14. La carpeta sobre la(s) encargada(s)/estafadora(s) estaba dentro de este armario grande.

(The folder on the manager(s)/scammer(s) was inside this large cabinet.)

15. El bolígrafo de este/estos contable(s)/pedante(s) estaba dentro de la oficina principal.

(The pen of this/these accountant(s)/pedant(s) was inside the main office.)

16. La alfombra para la(s) condesa(s)/criada(s) tenía pelo corto y muy brillante.

(The rug for the countess(es)/maid(s) was low pile and very shiny.)

17. La película con la(s) dama(s)/muda(s) era muy interesante y triunfó internacionalmente.

(The film with the lady(s)/mute(s) was very interesting and an international success.)

18. El dibujo con el/los caracol(es)/abejorro(s) estaba sin ninguna duda muy logrado.

(The drawing with the snail(s)/bumblebee(s) was without a doubt very successful.)

19. El gorro de aquel/aquellos mecánico(s)/cazador(es) era de gran colorido por seguridad.

(The hat of that/those mechanic(s)/hunter(s) was colorful for safety.)

20. La corbata con la(s) oveja(s)/abeja(s) sirvió para impresionar a los invitados.

(The tie with the sheep(∅)/bee(s) served to impress the guests.)

21. El video sobre el/los pavo(s)/ratón(es) provocó muy poco entusiasmo al principio.

(The video about the turkey(s)/mouse(s) elicited very little enthusiasm at first.)

22. El calcetín con el/los faisán(es)/chinche(s) llamaba la atención a primera vista.

(The sock with the pheasant(s)/bug(s) caught the eye at first glance.)

23. El cepillo para el/los burro(s)/toro(s) estuvo en el establo durante días.

(The brush for the donkey(s)/bull(s) was in the barn for days.)

24. El peine de aquel/aquellos soltero(s)/torero(s) era de plata y muy brillante.

(The comb of that/those bachelor(s)/bullfighter(s) was silver and very shiny.)

25. La cartera de la(s) decoradora(s)/proxeneta(s) tenía fotos y tarjetas de crédito.

(The wallet of the decorator(s)/pimp(s) had photos and credit cards.)

26. El contrato para el/los limpiador(es)/mercenario(s) era muy poco claro y complejo.

(The contract for the cleaner(s)/mercenary(s) was very unclear and complex.)

27. El número sobre el/los gorrión(es)/tiburón(es) era el más vendido del kiosko.

(The issue about the sparrow(s)/shark(s) was the best seller on the newsstand.)

28. La piedra de la(s) arqueóloga(s)/torturadora(s) estaba muy bien pulida y afilada.

(The stone of the archaeologist(s)/torturer(s) was very well polished and sharpened.)

29. La antena bajo la(s) gaviota(s)/urraca(s) estaba más bien vieja y doblada.

(The antenna under the gull(s)/magpie(s) was rather old and bent.)

30. La chaqueta de la(s) cocinera(s)/asesina(s) estaba dentro de la habitación pequeña.

(The jacket of the cook(s)/murderer(s) was inside the small room.)

31. La respiración de esta(s) buceadora(s)/esclava(s) era asombrosamente muy rítmica y pausada.

(The breathing of this diver(s)/slave(s) was surprisingly very rhythmic and slow.)

32. El monitor de este/estos topo(s)/cuervo(s) registraba muy precisamente el rítmo cardíaco.

(The monitor of this/these mole(s)/crow(s) recorded very precisely the heart rate.)

33. La cuchara con la(s) almeja(s)/babosa(s) estaba sobre la tabla de cortar.

(The spoon with the clam(s)/slug(s) was on the cutting board.)

34. El cheque para el/los compositor(es)/divorciado(s) estaba en la sala de reuniones.

(The check for the songwriter(s)/divorcé(s) was in the meeting room.)

35. La manta con la(s) salamandra(s)/garrapata(s) estaba mucho más limpia que ayer.

(The blanket with the salamander(s)/tick(s) was much cleaner than yesterday.)

36. La tabla con la(s) libélula(s)/polilla(s) mostraba exactamente donde estaba ese lugar.

(The table with the dragonfly(s)/moth(s) showed exactly where that spot was.)

37. La sábana sobre la(s) tortuga(s)/culebra(s) tenía un típico color marrón oscuro.

(The sheet over the tortoise(s)/snake(s) was a typical dark brown color.)

38. La cesta de la(s) trucha(s)/anguila(s) estaba a muy buen precio ayer.

(The basket of trout(s)/eel(s) was at a very good price yesterday.)

39. El artículo sobre el/los submarinista(s)/secuestrador(es) apareció en las noticias del periódico.

(The article about the submariner(s)/kidnapper(s) appeared in the newspaper news.)

40. El coche de este/estos anciano(s)/obispo(s) era de otra época y lento.

(The car of this/these elder(s)/bishop(s) was from another era and slow.)

41. La foto de la(s) gacela(s)/medusa(s) era muy impresionante por su colorido.

(The photo of the gazelle(s)/jellyfish(∅) was very impressive because of its color.)

42. La investigación sobre la(s) luciérnaga(s)/sanguijuela(s) atrajo el interés de mucha gente.

(The investigation for the firefly(ies)/leech(es) attracted the interest of many people.)

43. La imagen de esta(s) sepia(s)/pulga(s) causó ayer muchas discusiones y controversia.

(The image of this cuttlefish(∅)/flea(s) yesterday caused much discussion and controversy.)

44. El resumen de este/estos abogado(s)/soldado(s) superó las expectativas de los especialistas.

(The summary of this/these lawyer(s)/soldier(s) exceeded the expectations of the specialists.)

45. El trabajo con el/los médico(s)/ministro(s) era muy exigente y poco interesante.

(The work with the doctor(s)/minister(s) was very demanding and uninteresting.)

46. El capítulo sobre el/los fontanero(s)/verdugo(s) contaba detalles poco conocidos hasta ahora.

(The chapter on the plumber(s)/executioner(s) had little-known details until now.)

47. El uniforme para el/los herrero(s)/tirano(s) era de mala calidad y viejo.

(The uniform for the blacksmith(s)/tyrant(s) was of poor quality and old.)

48. El proceso sobre el/los jubilado(s)/político(s) progresó muy lentamente pero generó resultados.

(The process on the pensioner(s)/politician(s) progressed very slowly but produced results.)

49. La bolsa con la(s) garza(s)/mosca(s) está muy de moda en España.

(The bag with the heron(s)/fly(ies) is very fashionable in Spain.)

50. El documental sobre el/los hipopótamo(s)/murciélago(s) viajó por tres ciudades de África.

(The documentary about the hippopotamus(es)/bat(s) traveled through three cities in Africa.)

51. El plato con el/los reno(s)/sapo(s) tenía mucha fama en este país.

(The dish with the reindeer(s)/toad(s) was very famous in this country.)

52. El precio de este/estos dromedario(s)/escarabajo(s) era muy alto para su presupuesto.

(The price of this/these camel(s)/beetle(s) was too high for your budget.)

53. La herramienta de la(s) veterinaria(s)/sepulturera(s) estaba antes sobre la mesa grande.

(The tool of the vet(s)/grave digger(s) was on the big table before.)

54. El libro de ese/esos panadero(s)/sacerdote(s) explicaba cómo encontrar trabajo para

vivir.

(The book of that/those baker(s)/priest(s) explained how to find work for a living.)

55. El plan de este/estos informático(s)/delincuente(s) era pobre y muy poco elaborado.

(The plan of this/these computer scientist(s)/criminal(s) was poor and very little

elaborated.)

56. El monedero de este/estos pintor(es)/vago(s) estaba en el suelo medio vacío.

(The purse of this/these painter(s)/slacker(s) was half empty on the floor.)

57. El reportaje sobre el/los escultor(s)/pedófilo(s) recibió sorprendentemente varios

premios en Cannes.

(The report on the sculptor(s)/pedophile(s) surprisingly received several awards at

Cannes.)

58. El ensayo con el/los ingeniero(s)/gamberro(s) sirvió para determinar posibles estrategias

futuras.

(The rehearsal with the engineer(s)/thug(s) served to determine possible future

strategies.)

59. La cara de la(s) gimnasta(s)/traidora(s) revelaba con precisión todo su sufrimiento.

(The face of the gymnast(s)/traitor(s) accurately revealed all of her suffering.)

60. La casa de esta(s) notaria(s)/ladrona(s) estaba fuera del nuevo centro comercial.

(The house of this notary(s)/thief(ves) was outside the new shopping center.)

61. La leyenda sobre la(s) arqueóloga(s)/sepulturera(s) explicaba bastante bien lo que

sucedió.

(The legend about the archaeologist(s)/gravedigger(s) explained quite well what

happened.)

62. La cita con la(s) condesa(s)/drogadicta(s) era para poder entregarle el caballo.

(The appointment with the countess(es)/drug addict(s) was to deliver the horse.)

63. La plataforma para la(s) cigüeña(s)/urraca(s) estaba encima de este tejado verde.

(The platform for the stork(s)/magpie(s) was on top of this green roof.)

64. La charla con la(s) cocinera(s)/proxeneta(s) fue más interesante de lo esperado.

(The chat with the cook(s)/pimp(s) was more interesting than expected.)

65. La aventura de la(s) jardinera(s)/prostituta(s) avanzó muy rápidamente durante la

película.

(The adventure of the gardener(s)/prostitute(s) progressed very quickly during the film.)

66. La libreta de la(s) notaria(s)/muda(s) sirvió para poder aclarar algunas dudas.

(The notebook of the notary(ies)/mute(s) served to clarify some doubts.)

67. La sombra de la(s) cebra(s)/culebra(s) era mucho más nítida en fotografía.

(The shadow of the zebra(s)/snake(s) was much sharper in photography.)

68. La llamada de la(s) gacela(s)/hiena(s) alertó muy rápidamente a los animales.

(The call of the gazelle(s)/hyena(s) very quickly alerted the animals.)

69. La carpeta sobre la(s) arquitecta(s)/torturadora(s) estaba ayer en la oficina pequeña.

(The folder on the architect(s)/torturer(s) was in the small office yesterday.)

70. La alfombra con la(s) jirafa(s)/mofeta(s) estaba debajo de la mesa grande.

(The rug with the giraffe(s)/skunk(s) was under the big table.)

71. La película sobre la(s) almeja(s)/anguila(s) estaba antes junto a la pecera.

(The film about the clam(s)/eel(s) used to be next to the fish tank.)

72. La corbata de la(s) decoradora(s)/traidora(s) era bastante más colorida que aquella.

(The tie of the decorator(s)/traitor(s) was much more colorful than that one.)

73. La cartera de la(s) camarera(s)/criada(s) apareció al lado de este paragüero.

(The wallet of the waitress(es)/maid(s) appeared next to this umbrella stand.)

74. La piedra de la(s) buceadora(s)/asesina(s) sirvió para solucionar rápidamente el caso.

(The stone of the diver(s)/murderer(s) served to quickly solve the case.)

75. La antena de la(s) luciérnaga(s)/mosca(s) era antes más grande que ahora.

(The antenna of the firefly(ies)/fly(ies) used to be larger than it is now.)

76. La chaqueta de la(s) veterinaria(s)/esclava(s) era algo más pequeña que esta.

(The jacket of the vet(s)/slave(s) was somewhat smaller than this.)

77. La respiración de esta(s) oveja(s)/estafadora(s) era ahora más pausada que antes.

(The breathing of this sheep(∅)/swindler(s) was now more leisurely than before.)

78. La cuchara para la(s) dama(s)/ostra(s) estaba al lado del plato plano.

(The spoon for the lady(ies)/oyster(s) was next to the flat plate.)

79. La manta con la(s) tortuga(s)/pulga(s) llevaba mucho tiempo en esa esquina.

(The blanket with the tortoise(s)/flea(s) had been in that corner for a long time.)

80. La tabla sobre la(s) salamandra(s)/babosa(s) estaba ahí para evitar el barro.

(The board above the salamander(s)/slug(s) was there to avoid the mud.)

81. La sábana con la(s) libélula(s)/polilla(s) estaba mucho más vieja y agujereada.

(The sheet with the dragonfly(ies)/moth(s) was much older and had holes in it.)

82. La cesta con la(s) sepia(s)/tarántula(s) estaba muy bien tapada y vigilada.

(The basket with the cuttlefish(∅)/tarantula(s) was very well covered and guarded.)

83. La foto de la(s) gaviota(s)/garrapata(s) apareció hace poco en esta revista.

(The photo of the seagull(s)/tick(s) appeared recently in this magazine.)

84. La investigación sobre la(s) garza(s)/sanguijuela(s) fue hace poco publicada en Natura.

(Research on the heron(s)/leech(es) was recently published in Nature.)

85. La imagen de la(s) lechuza(s)/medusa(s) impresionó mucho a esta niña pequeña.

(The image of the owl(s)/jellyfish(∅) impressed this little girl very much.)

86. La bolsa con la(s) trucha(s)/víbora(s) estaba muy bien cerrada con cuerdas.

(The bag with the trout(s)/snake(s) was very well closed with strings.)

87. La herramienta de esta(s) encargada(s)/ladrona(s) apareció dentro de la caja fuerte.

(The tool of this manager(s)/thief(s) appeared inside the safe.)

88. La cara de la(s) foca(s)/madrastra(s) mostraba ayer mucha felicidad y emoción.

(The face of the seal(s)/stepmother(s) yesterday showed a lot of happiness and emotion.)

89. La casa de la(s) gimnasta(s)/abeja(s) estaba detrás de aquella pequeña colina.

(The house of the gymnast(s)/bee(s) was behind that little hill.)

90. El cuadro con el/los faisán(es)/tirano(s) desapareció hace poco de este museo.

(The picture with the pheasant(s)/tyrant(s) has recently disappeared from this museum.)

91. El proyecto sobre el/los hipopótamo(s)/murciélago(s) fue este año premiado varias

veces.

(The project on the hippopotamus(es)/bat(s) was awarded several times this year.)

92. El traje de este/estos mecánico(s)/pedante(s) era mucho más elegante y divertido.

(The costume of this/these mechanic(s)/pedant(s) was much more elegant and fun.)

93. El espejo de este/estos sastre(s)/gamberro(s) era algo más extravagante y moderno.

(The mirror of this/these tailor(s)/thug(s) was something more extravagant and modern.)

94. El archivo sobre el/los grillo(s)/tiburón(es) estaba en el ordenador de Ana.

(The file about the cricket(s)/shark(s) was on Ana's computer.)

95. El bolígrafo de este/estos profesor(es)/dictador(es) estaba dentro de un maletín negro.

(The pen of this/these teacher(s)/dictator(s) was inside a black briefcase.)

96. El dibujo con el/los caracol(es)/sapo(s) era bastante más bonito sin colorear.

(The drawing with the snail(s)/frog(s) was much prettier without coloring.)

97. El gorro con el/los burro(s)/toro(s) estaba en la tienda de souvenirs.

(The hat with the donkey(s)/bull(s) was in the souvenir shop.)

98. El video sobre el/los submarinista(s)/violador(es) fue muy sorprendentemente censurado

durante años.

(The video about the diver(s)/rapist(s) was very shockingly censored for years.)

99. El calcetín de este/estos pintor(es)/delincuente(s) apareció debajo de la cama grande.

(The sock of this/these painter(s)/delinquent(s) appeared under the big bed.)

100. El cepillo de aquel/aquellos limpiador(es)/viudo(s) era de madera de boj joven.

(The brush of that/those cleaner(s)/widower(s) was made of young boxwood.)

101. El peine de este/estos soltero(s)/político(s) estaba sobre el lavabo del baño.

(The comb of this/these bachelor(s)/politician(s) was on the bathroom sink.)

102. El contrato de aquel/aquellos peón(es)/mercenario(s) llegó ayer mediante correo certificado urgente.

(The contract of that/those pawn(s)/mercenary(ies) arrived yesterday by urgent certified mail.)

103. El número de este/estos contable(s)/preso(s) apareció muy rápidamente entre las pruebas.

(The number of this/these accountant(s)/inmate(s) came up very quickly between tests.)

104. El monitor de este/estos anciano(s)/vago(s) mostraba con claridad la actividad neuronal.

(The monitor of this/these elder(s)/slacker(s) clearly showed neural activity.)

105. El cheque para el/los abogado(s)/obispo(s) estaba dentro de la caja fuerte.

(The check for the lawyer(s)/bishop(s) was in the safe.)

106. El artículo sobre el/los topo(s)/chinche(s) era muy poco interesante y aburrido.

(The article about the mole(s)/bug(s) was very uninteresting and boring.)

107. El coche de aquel/aquellos panadero(s)/divorciado(s) estaba fuera de este pequeño taller.

(The car of that/those baker(s)/divorcé(s) was outside this small workshop.)

108. El resumen sobre este/estos escultor(s)/ministro(s) hablaba con detalle de su vida.

(The summary about this/these sculptor(s)/minister(s) spoke in detail of his/her life.)

109. El trabajo de aquel/aquellos fontanero(s)/verdugo(s) era muy poco agradable pero fácil.

(The work of that/those plumber(s)/executioner(s) was very unpleasant but easy.)

110. El capítulo sobre el/los pavo(s)/abejorro(s) era bastante más fácil de leer.

(The chapter on the turkey(s)/bumblebee(s) was a lot easier to read.)

111. El uniforme de este/estos luchador(es)/cazador(es) estaba dentro de aquel armario viejo.

(The uniform of this/these wrestler(s)/hunter(s) was inside that old cupboard.)

112. El proceso sobre el/los médico(s)/secuestrador(es) fue ayer bastante rápido y corto.

(The process regarding the doctor(s)/kidnapper(s) was quite quick and short yesterday.)

113. El documental sobre el/los herrero(s)/cuervo(s) impresionó ayer a todos los críticos.

(The documentary about the blacksmith(s)/raven(s) impressed all the critics yesterday.)

114. El plato de este/estos jubilado(s)/sacerdote(s) estaba bastante más lleno que aquel.

(The plate of this/these pensioner(s)/priest(s) was much fuller than that one.)

115. El precio de un/unos reno(s)/ratón(es) era algo más barato en Finlandia.

(The price of a/some reindeer(s)/mouse(s) was somewhat cheaper in Finland.)

116. El libro de aquel/aquellos compositor(es)/pedófilo(s) explicó con mucho detalle su biografía.

(The book of that / those composer(s)/pedophile(s) explained in great detail the biography of him.)

117. El plan de este/estos ingeniero(s)/soldado(s) facilitó que el rescate fuera exitoso.

(The plan of this/these engineer(s)/soldier(s) made it easier for the rescue to be successful.)

118. El monedero de este/estos informático(s)/torero(s) tenía en el dorso un dibujo.

(The purse of this/these computer scientist(s)/bullfighter(s) had a drawing on the back.)

119. El reportaje sobre el/los dromedario(s)/escarabajo(s) mostraba muy claramente su hábitat natural.

(The report about the dromedary(ies)/beetle(s) showed very clearly the natural habitat of it.)

120. El ensayo sobre el/los gorrión(es)/tábano(s) impresionó muy agradablemente a la profesora.

(The essay on the sparrow(s)/gadfly(ies) made a very pleasant impression on the teacher.)
